# Anxiety symptoms and their frequencies in albanian children: Differences by age, gender and other variables

**DOI:** 10.1192/j.eurpsy.2021.1684

**Published:** 2021-08-13

**Authors:** V. Skendi, V. Alikaj, E. Dashi

**Affiliations:** 1 Department Of Neuroscience, University Hospital Center “Mother Theresa”, Tirane, Albania; 2 Faculty Of Medicine, Tirana Medical University Albania, Tirane, Albania

**Keywords:** anxiety disorders, albanian children, Symptoms, childhood

## Abstract

**Introduction:**

Anxiety symptoms in childhood represent an important risk factor for developing anxiety disorders in subsequent developmental stages. This study examines the frequency and characteristics of the symptoms of the principal anxiety disorders in children and adolescents using a self-report questionnaire based on the diagnostic categories of the American Psychiatric Association (APA) manual.

**Objectives:**

Our main aim was to have a bigger view of anxiety symptoms spectrum in Albanian children, their frequencies and diferences related to age, gender or other variables.

**Methods:**

A cross-sectional, non-interventional study was conducted on 50 children/adolescents aged 8 to 17 years (45% males), frequenting Child/Adolescent Psychiatric Service, who completed the Spence Children’s Anxiety Scale.

**Results:**

More than one in four of the children and adolescents showed high scores in any anxiety disorder. The anxiety symptoms due to separation were the most frequent in the sample (5.5%), followed by physical fears. Girls scored significantly higher in all disorders (P < .001), except in obsessive-compulsive disorder. Differences were found as regards to age in all disorders, except physical fears, but the effect sizes were only in anxiety due to separation, which decreased with age, and generalized anxiety, which was higher in adolescents than in children.

**Conclusions:**

This study puts emphasizes to the early detection of anxiety symptoms in children, in order to provide the early and effective intervention and prevent the development of anxiety disorders in later life.

**Disclosure:**

No significant relationships.

